# Polyurethane Foams for Domestic Sewage Treatment

**DOI:** 10.3390/ma14040933

**Published:** 2021-02-16

**Authors:** Ewa Dacewicz, Joanna Grzybowska-Pietras

**Affiliations:** 1 Faculty of Environmental Engineering and Land Surveying, University of Agriculture in Kraków, Al. Mickiewicza 24/28, 30-059 Kraków, Poland; 2 Faculty of Materials, Civil and Environmental Engineering, Institute of Civil Engineering, The University of Bielsko-Biala, ul. Willowa 2, 43-309 Bielsko-Biała, Poland; jpietras@ath.bielsko.pl

**Keywords:** polyurethanes, waste foams, biofilter, physical properties, domestic sewage with low C/N ratio

## Abstract

The aim of the study was to assess the possibility of using polyurethane foams (PUF) as a filling of a foam-sand filter to directly treat domestic sewage with increased content of ammonium nitrogen and low organic carbon to nitrogen ratio (C/N). The study compared performance of two types of flexible foams: new, cylinder-shaped material (Novel Foams, NF) and waste, scrap foams (Waste Foams, WF). The foams serving as a filling of two segments of a foam-sand filter were assessed for their hydrophobic and physical properties and were tested for their cell structure, i.e., cell diameter, cell size distribution, porosity, and specific surface area. The study accounted also for selected application-related properties, such as hydrophobicity, water absorption, apparent density, dimensional stability, amount of adsorbed biomass, and the possibility of regeneration. Cell morphology was compared in reference foams, foams after 14 months of the filter operation, and regenerated foams. The experimental outcomes indicated WF as an innovative type of biomass carrier for treating domestic sewage with low C/N ratio. SEM images showed that immobilization of microorganisms in NF and WF matrices involved the formation of multi-cellular structures attached to the inner surface of the polyurethane and attachment of single bacterial cells to the foam surface. The amount of adsorbed biomass confirmed that the foam-sand filter made up of two upper layers of waste foams (with diameters and pore content of 0.50–1.53 mm and 53.0–63.5% respectively) provided highly favorable conditions for the development of active microorganisms.

## 1. Introduction

As one of the most versatile plastics, polyurethanes are widely used in industry and everyday life. They are commonly utilized e.g., in the construction industry (rigid foams for the production of insulation elements), in the furniture sector (foam filling upholstered furniture and comfort foam in mattresses), in the automotive industry (technical foam in car seats and molded foam in dashboards), as varnishes and polyurethane adhesives, in the production of leather materials (soles, haberdashery or clothing), and as packaging spacers.

The physical properties of polyurethane foam depend on the production method and the chemicals used. Properties such as foam density, cell structure, wetting rate, and water retention are modified by controlling the polymer to crosslinker ratio, foaming temperature, pH, and the type and amount of surfactant [1,2].

The polyurethane foam can be manufactured to have the desired properties as well as a sponge-like texture. The thin layer separating individual gas bubbles may be broken at the final expansion step, depending on the relative rate of molecular growth (gelling) and the gas reaction to produce a pore structure that is open (bubbles merge) or closed (bubbles do not merge). Open-cell foams are used, for example, in air filters, while closed-cell foams are popular insulating materials, e.g., in refrigeration appliances. The pores can be of different sizes and the foams can be enriched with numerous additives. Due to porosity, polyurethanes can be divided into polyurethane foams (porous products) and non-porous polyurethanes. The latter include massive polyurethanes, e.g., elastomers, flat polyurethanes, e.g., fabric coatings, or special polyurethanes, e.g., microcapsules used in medicine. In terms of cross linking density, the porous foams can be soft, i.e., flexible (fittings and blocks used in furniture cushions) or hard, i.e., rigid (panels with coating layers used in construction boards). Moreover, polyurethane foam can also be made of hydrophilic materials that can be easily rewetted in case of drying.

The main applications of polyurethane foams in environmental protection involve cultivation of microorganisms immobilized in PUF matrices [3,4,5,6], immobilization of active enzymes in the foam [1,7], biological treatment of exhaust gases, e.g., ammonia [8,9], and removal of NH_4_^+^ from industrial wastewater [10]. There are numerous research papers describing the ways microorganisms may colonize polyurethane foams in laboratory scale cultures. Typically, the biomass retained on a carrier comes in two different forms, one of which is a thick and dense biofilm that develops on the carrier surface, and the other is trapped in the pores of the carrier. Veresche et al. [11] investigated cubic PUF matrices as a biomass carrier and identified small multicellular structures attached to the inner surface of the polyurethane, single bacterial cells attached to the foam surface, and microgranules mechanically retained in its pores.

Contrary to the above, the literature search returns few reports on the use of polyurethane foam in biofiltration, and those that do address the subject usually do not provide detailed information on the properties of the filtering material. Shareefdeen et al. [12] investigated a biomass carrier made up of shredded polyurethane foam mixed with peat, perlite, and vermiculite. They concluded that the foam properties were not as conducive to biofiltration as those of the other tested materials. Without specifying the properties of PUR foam, they reported it as unfavorable for biofilm formation and therefore not contributing to the biofiltration efficiency.

In the 1980s, Japanese researches began investigating an interesting application of a sponge material made of flexible or hardened polyurethane. They developed a technology of an Upflow Anaerobic Sludge Blanket (UASB) reactor combined with a Down-flow Hanging Sponge (DHS) reactor for treatment of low-concentrated domestic or municipal wastewater [13,14,15,16,17,18,19,20,21,22,23,24,25]. Bundy et al. [26] investigated the use of a four-module reactor filled with foams with different pore contents (20 or 45 pores per inch) for direct purification of domestic wastewater containing N–NH_4_^+^ in an amount of approx. 30 mg/L. Jowett [27] suggested application of a “Waterloo biofilter”, filled with foam cubes serving as a biomass carrier, as a device for direct treatment of wastewater from summer lodgings and a golf course. According to the author, the contamination was deposited on the foam surface, which translated into long service life of the filter without significant decrease of the filtration parameters.

One of the most interesting biomass carriers are foams placed in a plastic structure, known under the trade name SuperBiomedia. They are used in trickling filters to improve the efficiency of existing bioreactors [28]. Hiep et al. [29] studied Bio-bact spheres of similar construction used in a DHS reactor for direct purification of domestic wastewater containing N–NH_4_^+^ in the amount of 40 ± 20 mg/L. However, foam fillings in the filters used for direct treatment of concentrated domestic sewage with low C/N ratio and ammonium nitrogen concentration exceeding 100 mg/L have not been widely investigated so far.

Polyurethane foam is harmless to the environment, which means it does not directly pollute our surroundings. It is also odorless and does not affect human physiology. Moreover, polyurethane foam does not rot and is pretty resistant to variable weather conditions. These properties determine the conditions for storing polyurethane foam waste that is particularly troublesome due to the low density of the material. The foam takes up a huge space and does not undergo biodegradation. It is therefore necessary, for both economic and environmental reasons, to develop methods for economic use of waste PUR foams.

One of the methods of their management is using them as a raw material for the manufacture of new products or as a substitute for the original raw material (material or raw material recycling). An example of managing the scraps of upholstery foams is using them as filling, for example, for pillows or toys. Waste foams can also be used for energy recovery.

This study evaluated the possibility of using spongy material in the form of flexible polyurethane foams as a carrier of microbial biomass in biofilters. The study involved determination of the hydrophobic and physical properties of unused (reference) and used foams (after 14 months of filter operation). Assessing the content of microorganisms adsorbed on flexible PUR foams allowed us to evaluate this type of material as a potential biomass carrier. The experimental outcomes indicated the relationship between the type of PUR foam and life span and stability of the biofilter operation and determined the effect of clogging on the decrease in the filter efficiency.

## 2. Materials and Methods

### 2.1. PUR Foams Used in the Study

The study used two groups of commercially available materials in the form of flexible PUR foams with a spongy structure. The first included brand new cylindrical foams measuring 2.5 cm × 13 cm (Novel Foams NF, Rossmann, Łódź, Łódź Province, Poland), while the second group involved waste scrap material (Waste Foams; WF, Eurofoam, Zgierz, Łódź Province, Poland). This material was a mixture of flexible PUR foam strips of irregular (random) shapes and dimensions from 3 to 35 mm [30].

According to the information provided by a manufacturer, the foams used in the automotive industry are both polyester and polyether ones with a stiffness of 1.2 to 5.8 kPa and an apparent density from 13.5 to 38.0 kg·m^−3^ [31]. The research by Bedla and Dacewicz [32] showed that PUR foam waste was characterized by nominal diameters d_10_ and d_60_ of 4 mm and 10 mm, respectively. The coefficient of grain-size uniformity d_60_/d_10_ was 2.5. More detailed description of waste foams is presented in the papers by Dacewicz [33], Dacewicz and Chmielowski [34], Dacewicz and Jurik [35], or Chmielowski et al. [36].

Table 1 presents the types and selected physical properties of a brand new material used in the study as a waste foams. These properties are provided by the manufacturer [31]. Apparent density of foams was performed according to ISO 845 Standard. Determination of stress-strain characteristics in compression was carried out according to ISO 3386-1 Standard.

### 2.2. Determination of PUR Foam Properties

The properties of PUR foams were tested at the Laboratory of Water and Wastewater Technology of the University of Agriculture in Kraków, Poland and the Laboratory of Geosynthetics and Textile Products of the University of Bielsko-Biała, Poland in accordance with the applicable European and Polish standards. The selected flexible PUR foams, both the reference ones (before the experiment was launched) and those retrieved after 14 months of the experiment (used), were subjected to the assessment of their hydrophobic and physical properties and microscopic examination. We also compared the structure of the reference foams, the foams after 14 months of the filter operation, and the regenerated foams. The regeneration was carried out by rinsing out the microorganisms and other impurities adsorbed inside and on the surface of the foams with distilled water. Before starting the tests, samples of similar size were selected from each type of WF foam. The dimensions of NF and WF foams were approximated and were 25 mm (diameter) and 10 mm (height). These samples were conditioned according to a standard procedure. The specimens of each NF and WF foams were kept for 24 h at standard atmospheres in accordance with ISO 291 (temperature 23 ± 2 °C, relative humidity 50 ± 10%).

Laboratory tests aimed at determining **hydrophobic properties** of the reference foams included:

- flotation test

This is a comparative method that does not provide a quantitative assessment of the degree of hydrophobicity. It consisted in placing NF and WF pieces matched for size on the surface of water in a beaker and observing them for 6 days. Hydrophobicity degree was assessed based on the time the foams remained on the water surface and the time they began to sink [37].

- water absorption (WA)

Water absorption of foams was carried out according to ASTM D570-98 (2018) Standard (Test Method for Water Absorption of Plastics—Twenty-Four Hour Immersion) and ISO 62. The conditioned specimens were weighted (m_s_) and immersed to a depth of 1 cm in distilled water for 24 h at a temperature of 23 ± 1 °C. Then the PUR foams were removed from water and its excess was absorbed by a filter paper. The foam samples were reweighed (m) and water absorption was calculated according to the formula:

WA = (m − m_s_)/m × 100 [%] (1)

**Physical properties** of NF and WF foams were determined based on the following tests:

- apparent density as per ISO 845 standard [38].

Apparent density (q_o_) is the mass of the dry material (m_s_) divided by its total volume, together with pores (V_p_). The apparent density is calculated with the formula:

(2) $$q_{o}\;=\;\frac{m_{s}}{V_{p}}\;[g\cdot {\mathrm{dm}}^{-3}]$$

- open porosity by hydrostatic method

The measurement consisted of weighing the sample of dry foam in air (m_s_), immersing it in water, weighing the sample in water (m_w_), saturating the sample with water, and then drying it and reweighing in air (m_n_). Open porosity (P_o_) was calculated using the formula:

P_o_ = [(m_n_ − m_s_)/(m_n_ − m_w_)] × 100 [%] (3)

### 2.3. Microscopic Examination

Microscopic examination with an electron microscope allowed us to determine cell morphology, porosity, and specific surface area of NF and WF foams.

***Morphology of PUR foam cells*** was determined with a scanning microscope (Jeol, JSM-5500 LV; JEOL Ltd., Tokyo, Japan/Lanameter MP-3 microscope). High-resolution images were taken with a cooled camera of a charge-coupled device (Photometrics model CH 250 charge-coupled device, Tucson, AZ, USA). Air-dry samples of flexible foams were cut into 1.5-cm-thick pieces using a microtome blade. Digital photos of the area 8.95 × 7.21 mm at 50× and 200× magnification were analyzed. Prior to the observation of 3D structures of the PUR foams, the samples were sprayed with gold using Jeol JFC 1200 ion coater (JEOL USA Inc., Peabody, MA, USA).

***Porosity and specific surface area*** of PUR foams were estimated with digital image analysis. The foam microstructure was examined under microscope equipped with HDCE-X3(E) camera (Bresser GmbH, Rhede, Münsterland, Germany), and several dozen pore photos were taken for each batch of foams. To determine cross-sectional area and the length of the pore circumference, the photos were subjected to appropriate transformations using ImageJ software. Measurements were performed for each image after applying an intensity threshold to distinguish between the individual pores. The resulting binary images were used to calculate the length of the circumference of individual pores and total length of the circumference of all pores in the known area of the analyzed samples. The fraction of each cross-section occupied by the open space (pores) and the fraction of each cross-section occupied by polyurethane were calculated for each image. The perimeter length per area was multiplied by the unit of thickness and the percentage of the constant cross-sectional area to obtain an estimate of the area per unit of volume [39].

Porosity was calculated by dividing the pore volume by the corresponding volume of the foams. The measurements were repeated five times.

***The open pore size distribution*** in the analyzed flexible PUR foams, determined by the minimum and maximum Feret diameter, was presented by Dacewicz [30].

### 2.4. Content of the Adsorbed Biomass and SRT Sludge Retention Time

The average amount of biomass remaining in the filter was measured for the two foam segments. Dry biomass determination involved measuring total suspended solids and organic suspended solids in the PUR foam samples. To this end, we compared the weight of the reference foams before the experiment with the weight of the foams after its completion (foams with adsorbed biomass).

As polyurethane foam may burn at the temperature usually applied for dry mass determination (105 °C), foam drying temperature was lowered to 60–70 °C, and the drying time was prolonged to 24 h. Organic suspended solids were determined as per standard methods in the sludge washed out of the foams during the regeneration. The regeneration was carried out by rinsing the microorganisms and other impurities adsorbed inside and on the surface of the foams with distilled water.

The sludge retention time (SRT) was calculated according to the following formula:

SRT = (S_I_ × V_I_ + S_II_ × V_II_)/Q × X (4)

where:

S_I_, S_II_—total average amount of the sediment that remained in the upper (I) and lower (II) foam segment;

V_I_, V_II_—the volume of foams in segment I and II;

Q—average volumetric flow rate (m^3^/d);

X—VSS concentration in the outflow (filtrate) from segment II.

### 2.5. Lab-Scale Model

Research on the use of brand new foams and waste foams for direct treatment of domestic sewage characterized by an increased content of ammonium nitrogen and a low C/N ratio, was carried out in two vertical flow filters. The structure of a single experimental filter was presented by Dacewicz [30,33,39,40] and Dacewicz et al. [35]. The filters were operated automatically with a time controller. The sewage of stable qualitative composition was dosed with peristaltic pumps that were turned on 24 times a day and supplied the volume adjusted to the set hydraulic load.

As the foam filling absorbs water, its weight increases and structural deformations may occur. To avoid this, the tested materials were placed in separate segments, and their working volume was as proposed by Dacewicz [40]. The upper and middle segment were filled with PUR foams to a total thickness of 60 cm. NF foams with a stiffening inside were arranged vertically in the filter (nine pieces per segment). WF waste material was placed randomly in both segments. The lower, third segment of each filter column was filled with a 30 cm layer of quartz sand with an equivalent diameter d_10_ = 0.32 cm. The filling material was supported with a grate installed at the bottom of each segment.

The study investigated raw domestic sewage collected from a public institution located in Kraków. The experiment was conducted for 14 months at variable concentrations of pollutants supplied to the foam-sand filters [30,39]. The average value of COD and ammonium nitrogen in raw sewage amounted to 200 mg O_2_/L and 150 mg/L, respectively, while the C/N ratio ranged from 0.4 to 4.8.

## 3. Results and Discussion

### 3.1. Hydrophobic Properties of the Foams

Laboratory tests on determination of hydrophobic properties included a preliminary assessment of the degree of the material hydrophobicity. Hydrophobicity of the foams was evaluated in the 6-day flotation test. In the WF group, the lowest degree of hydrophobicity was determined for flexible bluish-green, yellow-green, and orange foams (Table 2). Brand new NF foams with the smallest pores exhibited the lowest degree of hydrophobicity. We also found the hydrophobic properties of lilac waste foams similar to those of NF foams. The hydrophobic properties of WF foams deteriorated over time as indicated by their falling to the bottom of the beaker during the 6-day observation. Contrary to brand new foams, the more hydrophilic nature of waste foams may facilitate their rewetting.

The material roughness has a significant effect on the macroscopic contact angle for surfaces i.e., on their hydrophobicity. Dang-vu et al. [41] found that the roughness of a surface has a significant effect on the kinetics of liquid penetration into porous material. These authors reported that the penetration rate of liquids into porous material with a rough surface was over two-fold greater than that for porous materials with smooth surface. We found a similar relationship in our research (Table 2). The brand new cylindrical foams with low roughness were above twice more hydrophobic than bluish-green and yellow-green waste scrap material. It should be noted that WF materials have been mechanically shredded in irregular shapes and it was the reason for their higher roughness and hydrophobicity.

Table 3 presents water absorption by PUR foams. Waste PUR samples absorbed water with over 90% efficiency, while in the new NF foams this value reached only 55%.

### 3.2. Physical Properties of the Foams

The highest apparent density among waste foams was determined for bluish-green and lilac ones and the lowest for orange foams with the greatest pores. Mean apparent density for four investigated WF foams was 13.67 g/L [42], while for the new foams it was 10.0 g/L.

Using the hydrostatic method for measuring open porosity of the foam, we determined the porosity of the new PUR NF at 79.5%. (Table 3). Waste bluish-green, yellow-green, and orange foams reached even higher porosity, similar to a value of 94.6% reported by other authors [35,42]. These porosity values were similar to values described by Yaya Beas et al. [43]. In their research, the new foams were saturated with distilled water in a beaker, removed from the beaker, and the remaining volume of water was measured. Yaya Beas et al. [43] found that the porosity of the new foams in the form of cubes was 90%.

We determined that the WA properties depend both on the morphology of PUR foams and their hydrophobic character. Due to the more open-cell structure of PUR foams, the water absorption properties increased. The high relation (r = 0.99) between open porosity and water absorption properties of NF and WF foams was observed. The increase in hydrophobic character of the tested foams caused a decrease in their water absorption. In this case, the correlation coefficient for tested foams was lower (r = 0.73).

### 3.3. Microscopic Evaluation of the Foams

#### 3.3.1. Cell Morphology of PUR Foams

**Figure 1:**
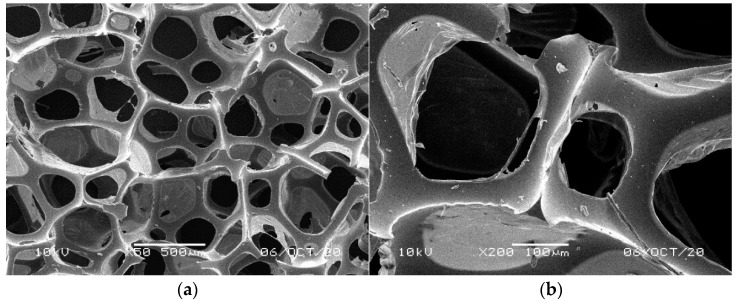
SEM images of the matrix of foam cells in brand new reference NF foams: (**a**) magnification 50×, (**b**) magnification 200×.

**Figure 2:**
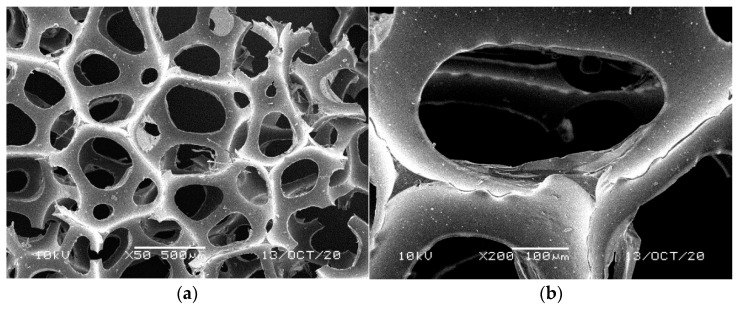
SEM images of the matrix of foam cells in reference bluish-green waste WF foams: (**a**) magnification 50×, (**b**) magnification 200×.

**Figure 3:**
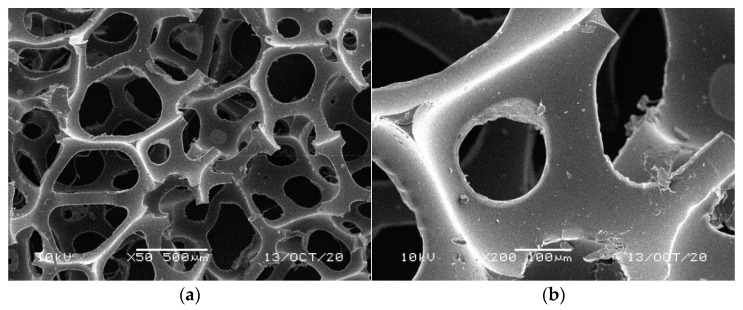
SEM images of the matrix of foam cells in reference yellow-green waste WF foams: (**a**) magnification 50×, (**b**) magnification 200×.

**Figure 4:**
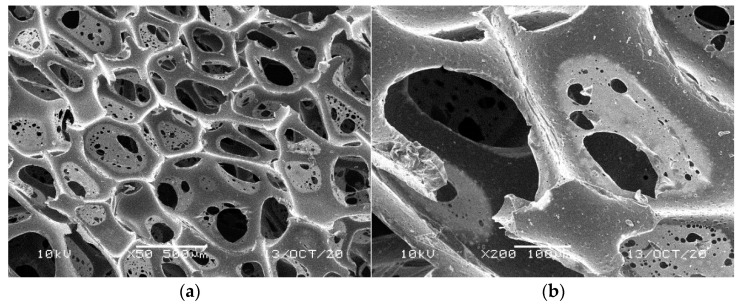
SEM images of the matrix of foam cells in reference lilac waste WF foams: (**a**) magnification 50×, (**b**) magnification 200×.

**Figure 5:**
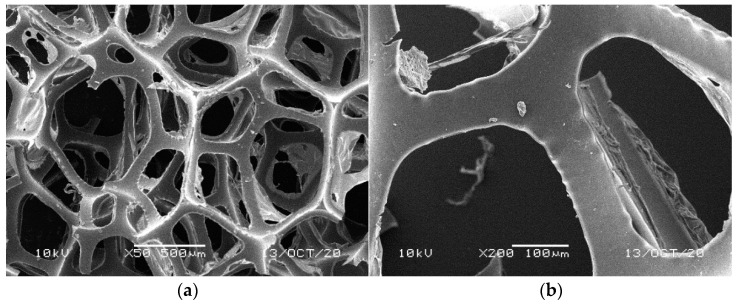
SEM images of the matrix of foam cells in reference orange waste WF foams: (**a**) magnification 50×, (**b**) magnification 200×.

Figure 1, Figure 2, Figure 3, Figure 4 and Figure 5 illustrate the reference PUR foams used in the study. The analyzed material had both a horizontal and vertical structure, which ensured the internal transfer of sewage and gases. The individual pores were mostly composed of pentagonal walls forming open cells of variable size and shape. They were separated by ribs formed at the contact point of at least three cells. The ribs reinforced the foam by affecting its stiffness and sensitivity to deformation. The pore shape factor, which denotes the object elongation as described by Dacewicz [30], amounted to 0.7 in NF foams. This means that the pore shape in the new foams was almost spherical (Figure 1). Lilac-colored WF foams turned out to be the thinnest and most elongated ones (Figure 4), as their Feret diameters were the smallest and the pore shape factor reached 0.54. This indicated that as a result of previous use, the lilac-colored foam cells were the most flattened. They also featured lowered stiffness, and their manufacturer classified this group of foams as comfort ones [31].

SEM analysis of the reference NF foams revealed lower roughness of their surface. The waste foams had more uneven surfaces due to pitting and small surface irregularities. These observations confirm the experiences of other authors. Bolton et al. [44], who conducted a lab-scale research of 14 different biomass carriers to be used in sewage treatment, found out that surface roughness favored the accumulation of biofilm. According to these authors, the rough material provided not only a larger area for biomass growth, but also sheltered sites for its anchoring that prevented its washing away from the carrier.

#### 3.3.2. Pore Size, Porosity and Specific Surface Area

The Feret diameters determined by Dacewicz [30] and the number of pores in NF and WF foams against their percentage content showed that the brand new foams featured the smallest pore diameter, on average 0.44 mm. The pore diameter of the waste foams ranged from 0.50 mm (the least rigid lilac foams) to 0.67 mm (the most rigid yellow-green foams).

The percentage of foams used in the experiment and the pore content of the samples, determined as per Feret diameters [30], are summarized in Table 4.

The porosity measured by digital image analysis [30] turned out to be lower than the open porosity determined by the hydrostatic method. The open pore content of the NF foams averaged 75.3%, while the pore content of the WF foams ranged from 53.0% to 63.5%. The values provided in the literature are higher, but many authors do not provide a method for determining porosity, and some rely on the values provided by the material manufacturer. Uemura et al. [45] experimented with a DHS reactor filled with foams of diameter and porosity specified by the manufacturer as 0.5 mm and 98%. Okubo et al. [21] found the empty space in new foams used for urban wastewater treatment to be at least 95%. Tawfik et al. [15] treated gray wastewater using new foams with a diameter of 0.63 mm and a high porosity index of 90%. In a study on foams in the form of cuboids and sheets, Yaya Beas et al. [43] assessed their porosity at 90% and 47%, respectively. Członka et al. [37] determined the porosity of polyurethane foams filling a biofilter to be 85%.

Digital image analysis indicated the average specific surface area of brand new cylindrical foams to be approximately 490 m^2^/m^3^. For the waste foams, the calculated surface area ranged from 266 m^2^/m^3^ (lilac foams) to 619 m^2^/m^3^ (bluish-green foams). These values are comparable to the surface area of the polyurethane filling described by Członka et al. [37] and the foam material used in DHS reactors. In their study on gray wastewater, Tawfik et al. [15] reported a specific surface area of 256 m^2^/m^3^ for PUR foams of pore diameter 0.63 mm and a density of 30 g/L. According to Onodera et al. [19], specific surface area of sixth generation foams with pore diameter of 1.6 mm was about 267 m^2^/m^3^. Uemura et al. [46] experimented with a DHS reactor filled with three types of foam filling with specific surface area within 205–217 m^2^/m^3^ range. Specific surface area of bluish-green and yellow-green waste material is comparable to the values of commercially available new generation media. The manufacturer of SuperBiomedia carriers, consisting in foams encased in a plastic frame, states that their specific surface area is 600 m^2^/m^3^ [28]. Also the specific surface area of the carrier material used in the Zander Biotrickling technology, based on employing polyurethane foam blocks as a biomass carrier in sprinkled beds (biotrickling), is determined to be approx. 600 m^2^/m^3^ with its apparent density approx. 20 kg/m^3^ [47].

#### 3.3.3. Content of the Adsorbed Biomass and Sludge Retention Time (SRT)

Table 5 summarizes the biomass content in flexible PUR foams used in the study against their pore diameter. It is important that the foams had high pore content and size, as they provide room for the growth and development of microorganisms without large pressure loss in the filter. Compared to the waste foams, the brand new cylindrical foams featured a higher percentage of pores and pores with the smallest Feret diameter (Table 3 and Table 4). These properties ensured adequate space for the microbial growth, and the biomass concentration at the level of 4 g/L of the foams in the second segment constituted 62.2% of the total suspension retained in this part of the filter.

**Figure 6:**
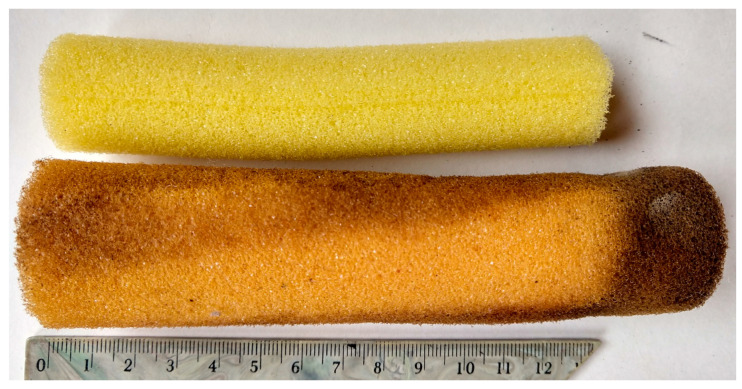
NF foams before research (at the top) and after 7 months of research (on the bottom).

The amount of biomass retained in filters filled with brand new cylindrical foams was considerably higher than in the case of waste foams of random shapes. The only exception was the least rigid lilac waste foams with the smallest pores. Machdar et al. [25], who studied foams of different pore size (0.56 to 1.92 mm) filling a DHS reactor, found out that the smaller the pore diameter, the more room for the microorganisms to attach. Dacewicz [39] pointed out that intense biomass growth in brand new, flexible PUR foams made the pores clog, and thus the specific area of the foams decreased. High porosity of the filler allowed for its efficient penetration and appropriate contact between the substrates and the population of active microorganisms. However, less rough surface of NF foam was not conducive to proper anchoring of the expanded biofilm and did not prevent its washing out of the carrier. As reported by Dacewicz [39], the excessive biomass accumulated in the lower part of the cylinders (Figure 6) was washed away after 7 months of research. Moreover, the layer of sand in the third segment was covered with a surface scum that finally caused clogging of the filter.

**Figure 7:**
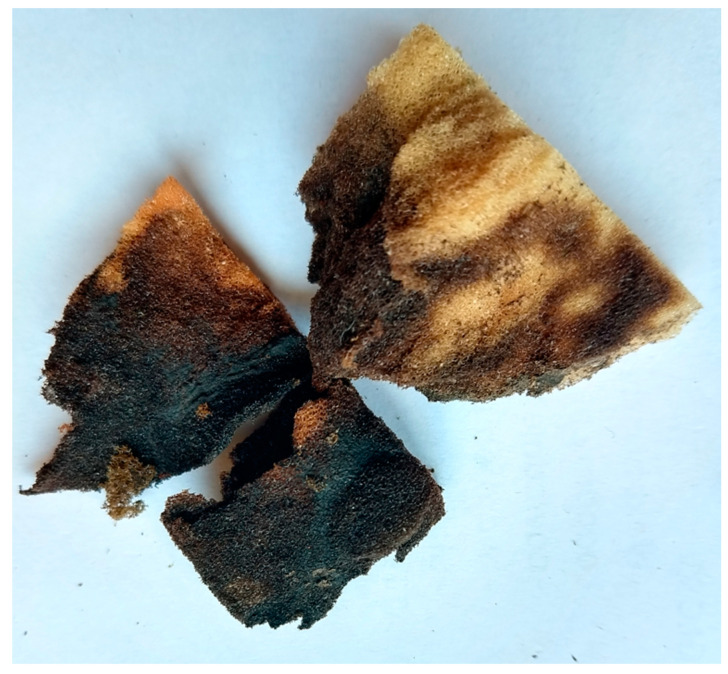
Excessive accumulation of microorganisms in the second segment of lilac WF foams.

Intense reproduction of microbial biomass was also found for lilac WF foams. Excessive accumulation of microorganisms blocked the pores, as confirmed by very high biomass concentration in the second segment, amounting to almost 5 g/L of the foams. The dimensional stability of the least rigid lilac foams also changed visibly (Figure 7). Scanning photos of the reference WF foams indicated a peculiar structure of a film-like nature in the pores of lilac foams (Figure 4), which could be responsible for greater biomass retention capacity of this waste material.

In a filter filled with waste foams, the bluish-green ones turned out to be the most suitable biomass carrier. Microorganism concentration of 1.7 g/L of the foams in the second segment, which constituted 87% of the total suspension retained in this part of the filter, did not contribute to pore blocking. The use of foams with a pore diameter of 0.62 mm prevented washing the biomass out of the second segment of the filter, and thus limited the concentration of organic suspension supplied to the bottom sand layer in the third segment. More information on this subject can be found in the author’s previous publications [30,39].

Different processes of biomass formation occurred at different levels of the filter (Table 5). The highest, over 40% increase in the percentage of biomass in individual segments, was observed in the case of waste bluish-green and lilac foams. No such visible microbial growth was noted for yellow and orange waste foams. After 14 months of the filter operation, the biomass content adsorbed on the foams of the second segment reached about 0.3 g/L. This value is similar to that given by Chmielowski et al. [48]. These authors reported that after 3 months of operation of a filter filled with 60 cm of flexible PUR foam scraps, the average biomass content in the mixture of foams was 0.1674 g/L of the filling, which was less than 10% of all retained impurities (TSS = 1.87 g/L of the applied material).

Other authors observed significantly higher values while testing the UASB-DHS system for municipal wastewater treatment. Okubo et al. [21] determined the total mean amount of sludge remaining in the DHS reactor at 36.3 ± 6.9 g VSS/L in the upper layer and at 27.6 ± 4.8 g VSS/L in the lower layer. Considering the total volume of the reactor foam filling, they estimated that the total amount of sludge in the reactor reached 966 kg VSS. Uemura and Harada [49] determined dry biomass content in a DHS reactor filled with foams of 0.5 mm diameter at the level of 20–30 g/L of the foams. Tawfik et al. [15], who studied direct treatment of gray wastewater, evaluated the amount of the accumulated biomass at 16–78 g/L of foams of 0.63 mm diameter.

SRT calculated according Formula 4 for foam filters turned out to be extremely long and amounted to approx. 200 days for waste foams, and 170 days for new foams. Such a long SRT was affected by a very low VSS concentration in the outflow from segments II. Other authors observed lower SRT values but for municipal wastewater treatment in the UASB-DHS system. Okubo et al. [21] determined SRT for a DHS reactor at about 69 days and Onodera [20] at about 90 days. These results indicate that the DHS process can be 10–20 times longer than the conventional activated sludge process (ASP) based on aerobic growth of microorganisms. According to the authors, the main reason for this relative drop in the sludge production was the longer retention time of the SRT sludge. It provided more opportunities for the autolysis of the retained sludge [50], and thus limited the growth of excess sludge typical of DHS reactors.

#### 3.3.4. Structural Properties of the Foams with Biomass

**Figure 8:**
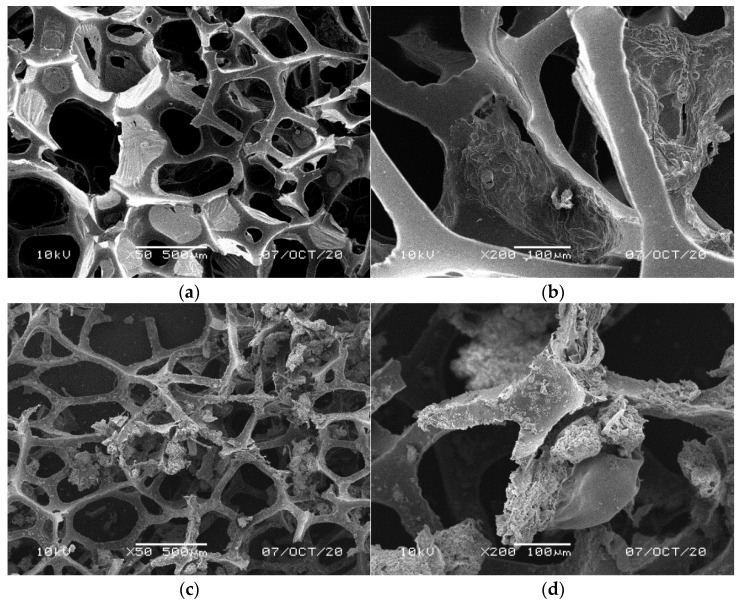
SEM images of accumulated sludge in the matrix of foam cells of new reference NF foams in the upper part of the cylinders (**a**) on the foam surface (**b**) and inside the foam; in the lower part of the cylinders (**c**) on the foam surface (**d**) and inside the foam.

**Figure 9:**
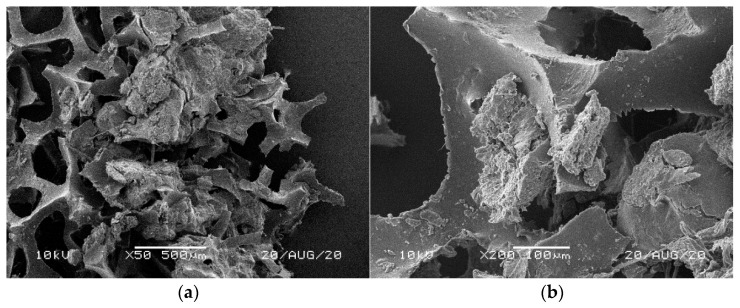
SEM images of accumulated sludge in the matrix of foam cells of waste reference bluish-green WF foams (**a**) on the foam surface (**b**) and inside the foam.

**Figure 10:**
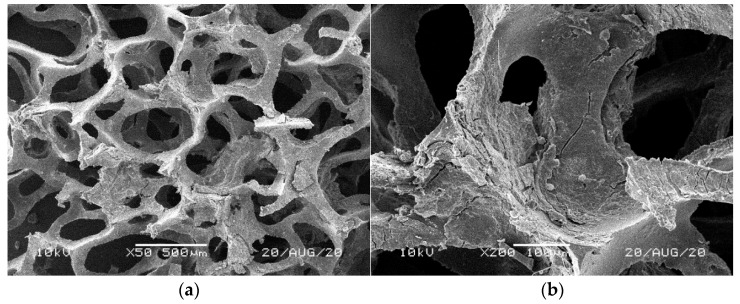
SEM images of accumulated sludge in the matrix of foam cells of waste reference yellow-green WF foams (**a**) on the foam surface (**b**) and inside the foam.

**Figure 11:**
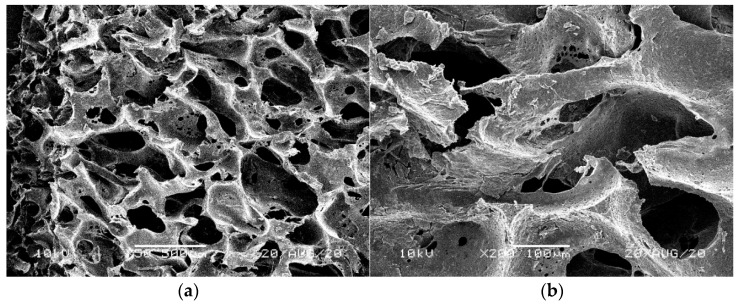
SEM images of accumulated sludge in the matrix of foam cells of waste reference lilac WF foams (**a**) on the foam surface (**b**) and inside the foam.

**Figure 12:**
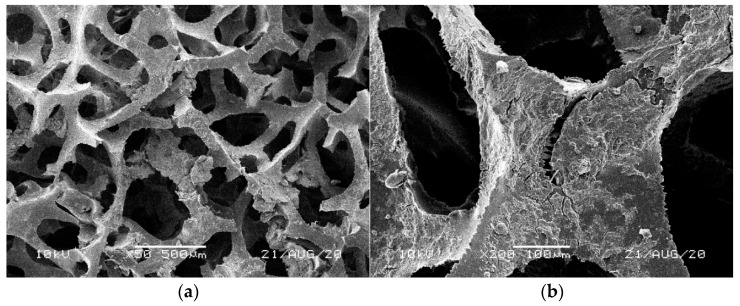
SEM images of accumulated sludge in the matrix of foam cells of waste reference orange WF foams (**a**) on the foam surface (**b**) and inside the foam.

Structural examination revealed significant differences between the surface of PUR foams collected from different levels of the filters. The control filter, filled with the new cylindrical foams, showed distinct multicellular structures attached to the inner surface of the polyurethane with typical fluffy surface (Figure 8). A fluffy structure of retained biomass was observed by Tawfik et al. [51] after 1 and 3 years of operation of an anaerobic hybrid reactor followed by downflow hanging sponge system treating domestic wastewater. Similar structures we observed for the biomass formed on the surface and inside the waste bluish-green foams (Figure 9). The remaining waste materials, i.e., yellow (Figure 10), lilac (Figure 11), and orange foams (Figure 12) had a smoother surface. In the lilac foams, where the number of microorganisms was the highest, the biomass was immobilized in the pores and also filled the spaces in the structure of foam cells (Figure 11a,b). Another type of microbial immobilization in cubic PUR foam matrices described by Veresche et al. [11] included single cells attached to the surface of individual foam pieces. Araki et al. [52] analyzed cube-shaped polyurethane foams and found nitrifying bacteria in their internal spaces but not in the biofilm attached to their surfaces. Guo et al. [53] investigated different levels of foam thickness in a biofilter with 100% recycling system and concluded that one centimeter of the foam was enough to effectively treat synthetic domestic wastewater with extremely high C/N ratio of around 20 (N–NH_4_^+^ 17–20 mg dm^−3^, COD 350–400 mg O_2_ dm^−3^). This thickness of the foam was determined as optimal for the growth of active microorganisms, both inside and on its surface.

SEM analysis of the foams showed clear differences in the colonization of cylindrical NF foams. Their upper parts (Figure 8a,b) harbored considerably lower amount of the microorganisms than the lower ones (Figure 8c,d). This was caused by faster drying of the upper parts, which exhibited the highest hydrophobic properties. We also observed that after 14 months of the filter operation NF polyurethane foam irreversibly increased its size by 20% due to swelling. Figure 6 shows contact spots on the surface of two adjacent cylinders. These spots and the lower parts of the cylinders provided the most humid conditions conducive to the microbial growth. Empty space, initially filled with air, was later on colonized by microorganisms. As reported by Dacewicz [39], the excessive biofilm was washed out at the highest hydraulic load, and as a result, the sand layer clogged after 215 days of the filter operation. The filter was regenerated and made operational again by prompt removal of the clogging layer.

#### 3.3.5. Regeneration Process

In her previous papers, Dacewicz demonstrated that a filter filled with a mixture of waste PUR foams effectively treated domestic sewage with high content of ammonium nitrogen and low C/N ratio. The author comprehensively discussed her findings and compared them meticulously with literature data in two recently published papers [30,39]. For this reason, the last step of this study was a regeneration of more dimensionally stable waste PUR foams.

**Figure 13:**
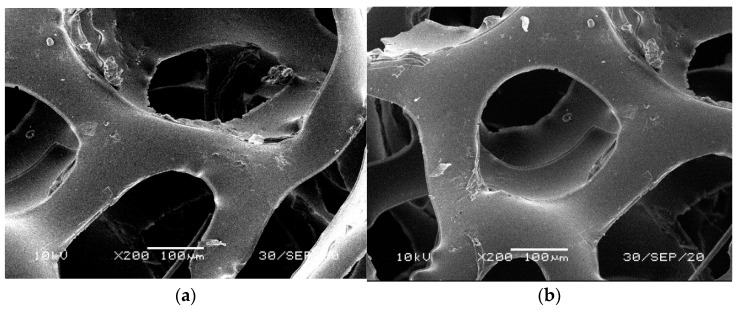
SEM images of the matrix of foam cells in bluish-green waste WF foams subjected to regeneration; magnification 200×, (**a**) inside the foam (**b**) and on the foam surface.

**Figure 14:**
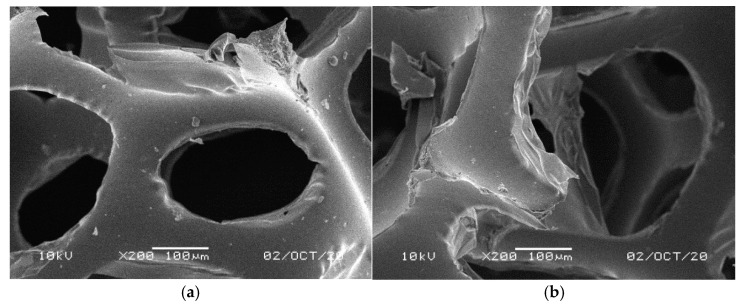
SEM images of the matrix of foam cells in yellow-green waste WF foams subjected to regeneration; magnification 200×, (**a**) on the foam surface (**b**) and inside the foam.

**Figure 15:**
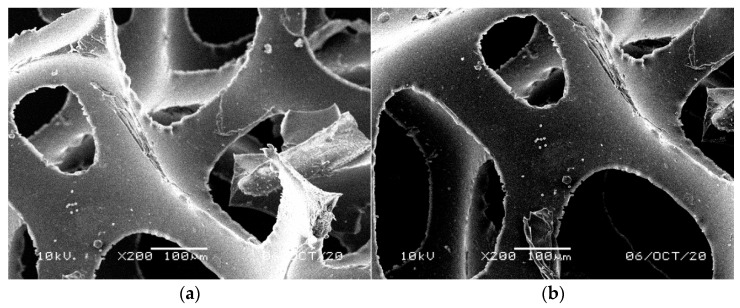
SEM images of the matrix of foam cells in orange waste WF foams subjected to regeneration; magnification 200×, (**a**) inside the foam (**b**) and on the foam surface.

**Figure 16:**
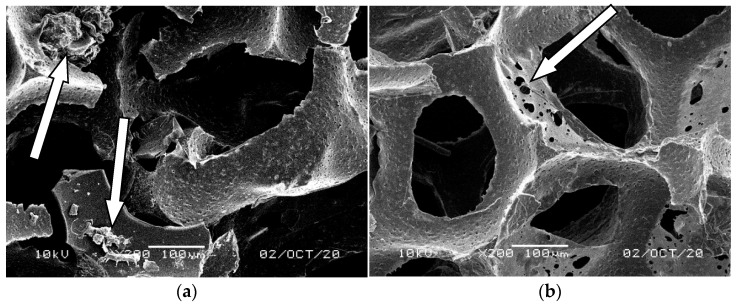
SEM images of the matrix of foam cells in lilac waste WF foams subjected to regeneration; magnification 200× (the arrows indicate unremoved biomass and pollutants inside the pores and on the surface of the foam skeleton), (**a**) inside the foam (**b**) and on the foam surface.

SEM analysis revealed intense washing out of the biomass and other impurities during regeneration from bluish-green (Figure 13), yellow-green (Figure 14), and orange foams (Figure 15). The regeneration was the least effective in lilac foams that had the smallest pores and where the biomass growth was the greatest (Table 4). Figure 16 clearly shows areas inside the pores and on the surface of the foam skeleton with multicellular fluffy structures of microorganism (Figure 16a) and other pollutants (Figure 16b) still attached to them.

## 4. Summary and Conclusions

The study indicated PUR foams as effective biomass carriers in a foam-sand filter employed for direct treatment of domestic sewage characterized by a high content of ammonium nitrogen (150 mg/L) and low organic carbon to nitrogen ratio (C/N).

Porosity, hydrophilicity, and shape of PUR foams affected not only the type of biomass but also the degree of clogging of the spongy material surface. The pore size of the material directly correlated with the amount of accumulated biomass. Another important factor while choosing the biomass carrier was water absorption capacity, as the microbial growth was the most intense in permanently moist parts of the foams.

Both the stiffest new foams with a pore diameter of 0.44 mm and pore content of 75.3%, and the least stiff waste foams with a pore diameter of 0.50 mm and pore content of 53.0% showed the greatest retention capacity for microorganisms. After 14 months of operation, the biomass concentration was 4 and 5 g/L of foams, respectively. On the one hand, retaining large amounts of biomass on the foam filling may facilitate self-degradation of the sludge, which can further reduce the excessive biomass production. The SRT calculated for the investigated filters was approximately 200 days and was about 40 times longer than that of the conventional activated sludge process. On the other hand, intense growth of biomass results in clogging small pores and formation of so-called dead zones in the filtration process caused by irreversible clogging of the spongy filling. The amount of biomass accumulated on the waste foams of pore diameter greater than 0.6 mm and pore content of about 60% turned out to be lower. In their case, the biomass concentration of 3 g/L did not cause the pores or the biofilter to clog.

SEM analysis showed that the microorganisms were retained on the carrier in the form of a fluffy biofilm that developed on the carrier’s surface and in a trapped form in its empty spaces.

Following the foam regeneration, single bacterial cells remained on the carrier surface only in the case of lilac waste foams, characterized by the lowest dimensional stability and porosity. To confirm the possibility of regenerating waste foams, further studies are planned with the use of regenerated foams for direct treatment of domestic sewage with a low organic carbon to nitrogen ratio.

## Figures and Tables

**Table 1:** Types and physical properties of foams as specified by Eurofoam Polska Sp. z o.o. [31].

Type	Content in the Mixture [%]	Apparent Density [kg m^−3^]	Compressive Strength * [kPa]	Basic Color
N 1418	3.8	13.5–15.5	1.6–2.3	white
N 1819	3.7	16.0–19.0	1.2–2.0	white
N 2030	16.5	17.0–21.0	2.7–3.6	white
N 2121	27.5	19.5–22.5	1.8–2.6	yellow-green
N 2538	8.6	22.0–26.0	3.3–4.6	lilac
N 2838	4.2	24.0–28.0	3.5–4.5	orange
N 3030	21.6	27.5–31.0	2.7–3.7	bluish-green
N 3543	8.5	32.0–36.0	3.8–5.0	bluish-green
N 3050	3.0	27.0–31.0	4.4–5.8	bluish-green
N 3843	2.6	34.0–38.0	4.0–5.3	blue

* Covers the determination of material with a density up to 250 kg/m^3^.

**Table 2:** Results of the 6-day flotation test of PUR foams.

Foam Color	Time After Which the Foam Is Fully Immersed [h]	Time After Which the Foam Sinks [h]
Brand new cylindrical foams (NF)		
Green	>144	>144
Waste foams of random shapes (WF)		
Bluish-green	16	72
Yellow-green	16	56
Lilac	80	130
Orange	24	104

**Table 3:** Water absorption, apparent density, and open porosity of PUR foams.

Foam Color	Water Absorption [%]	Apparent Density [g/L]	Open Porosity [%]
Brand new cylindrical foams (NF)			
Green	55.0 ± 1.16	10.0 ± 0.22	79.5 ± 1.83
Waste foams of random shapes (WF)			
Bluish-green	95.5 ± 2.24	13.62 ± 0.27	95.00 ± 2.64
Yellow-green	95.4 ± 2.25	15.89 ± 0.33	95.05 ± 2.66
Lilac	89.0 ± 2.23	15.44 ± 0.36	93.38 ± 2.80
Orange	95.6 ± 2.29	9.73 ± 0.21	94.91 ± 2.75

**Table 4:** Percentage of flexible PUR foams and their pore content [30].

Foam Color	Content in the Mixture [%]	Foam Pore Content [%]	Surface Area [m^2^/m^3^]
Brand new cylindrical foams (NF)			
Green	38.9	75.3 ± 2.1	487 ±17.6
Waste foams of random shapes (WF)			
Bluish-green	33.1	62.6 ± 1.9	619 ± 24.8
Yellow-green	27.5	63.5 ± 3.5	602 ± 34.9
Lilac	8.6	53.0 ± 2.9	266 ± 13.3
Orange	4.2	61.3 ± 5.5	552 ± 46.9

**Table 5:** Feret diameters and biomass content in flexible PUR foams used in the study.

Foam Color	Feret Diameter [mm]	Total Suspended Solids I/II Segment [g/L foam]	Biomass Content I/II Segment [g/L foam]	Percentage Biomass Content [%]
Brand new cylindrical foams				
Green	0.44 ± 0.01	0.1792	0.1115	62.2
Waste foams of random shapes				
Bluish-green	0.62 ± 0.03	0.154/1.912	0.063/1.663	40.9/87.0
Yellow-green	0.67 ± 0.04	0.0929/0.305	0.056/0.245	60.3/80.3
Lilac	0.50 ± 0.02	0.2300/5.667	0.1067/4.867	46.4/85.9
Orange	1.53 ± 0.012	0.0643/0.4114	0.0371/0.333	57.7/80.9

## Data Availability

The data presented in this study are available on request from the corresponding author.
